# Modeling of *FAN1*-Deficient Kidney Disease Using a Human Induced Pluripotent Stem Cell-Derived Kidney Organoid System

**DOI:** 10.3390/cells12182319

**Published:** 2023-09-20

**Authors:** Sun Woo Lim, Dohyun Na, Hanbi Lee, Xianying Fang, Sheng Cui, Yoo Jin Shin, Kang In Lee, Jae Young Lee, Chul Woo Yang, Byung Ha Chung

**Affiliations:** 1 Transplantation Research Center, College of Medicine, The Catholic University of Korea, Seoul 06591, Republic of Korea; 2 Division of Nephrology, Department of Internal Medicine, Seoul St. Mary’s Hospital, The College of Medicine, The Catholic University of Korea, Seoul 06591, Republic of Korea; 3 ToolGen, Inc., Seoul 06591, Republic of Korea

**Keywords:** *FAN1* gene, induced pluripotent stem cells, kidney organoid, karyomegalic interstitial nephritis

## Abstract

Karyomegalic interstitial nephritis (KIN) is a genetic kidney disease caused by mutations in the FANCD2/FANCI-Associated Nuclease 1 (*FAN1*) gene on 15q13.3, which results in karyomegaly and fibrosis of kidney cells through the incomplete repair of DNA damage. The aim of this study was to explore the possibility of using a human induced pluripotent stem cell (hiPSC)-derived kidney organoid system for modeling *FAN1*-deficient kidney disease, also known as KIN. We generated kidney organoids using WTC-11 (wild-type) hiPSCs and *FAN1*-mutant hiPSCs which include KIN patient-derived hiPSCs and *FAN1*-edited hiPSCs (WTC-11 ^FAN1+/−^), created using the CRISPR/Cas9 system in WTC-11-hiPSCs. Kidney organoids from each group were treated with 20 nM of mitomycin C (MMC) for 24 or 48 h, and the expression levels of Ki67 and H2A histone family member X (H2A.X) were analyzed to detect DNA damage and assess the viability of cells within the kidney organoids. Both WTC-11-hiPSCs and *FAN1*-mutant hiPSCs were successfully differentiated into kidney organoids without structural deformities. MMC treatment for 48 h significantly increased the expression of DNA damage markers, while cell viability in both *FAN1*-mutant kidney organoids was decreased. However, these findings were observed in WTC-11-kidney organoids. These results suggest that *FAN1*-mutant kidney organoids can recapitulate the phenotype of *FAN1*-deficient kidney disease.

## 1. Introduction

Karyomegalic interstitial nephritis (KIN) is a rare hereditary cause of chronic interstitial nephritis. Previous studies have found that KIN is ascribed to autosomal recessive mutations in the *FAN1* (FANCD2/FANCI-Associated Nuclease 1) gene, which is a downstream effector of the Fanconi anemia (FA) DNA repair pathway [1,2]. *FAN1* deficiency results in DNA inter-strand cross-link (ICL) repair defects, finally enhancing tissue karyomegaly and organ dysfunction as shown in KIN [3,4,5,6,7]. Interestingly, *FAN1* protein is highly expressed in the kidney, suggesting that the kidney might depend on *FAN1* for maintaining its normal function [1]. In addition, it has been suggested that chromosomal instability caused by a failure in DNA repair in KIN can be one of the important mechanisms underlying chronic kidney disease (CKD) [1]. Indeed, recent studies have unveiled the mechanism by which a defect in ICL repair leads to the progression of CKD using *FAN1*-KO mice and human tubular epithelial cells [1,2,8].

Meanwhile, human induced pluripotent stem cells (hiPSCs) can be dedifferentiated from somatic cells including skin fibroblasts, adipose cells, and peripheral mononuclear cells (PBMCs) [9,10,11,12]. The most important feature of hiPSCs is that they can differentiate into diverse types of somatic cells or organoids [13,14,15]. Additionally, genetically diverse hiPSCs can be created by using patient-derived somatic cells or by performing gene editing using the CRISPR/Cas9 system [16,17]. Levering these characteristics of hiPSCs, creating a disease model through the differentiation of genetically diverse hiPSCs into kidney organoids could represent a breakthrough for modelling various hereditary kidney diseases. Indeed, previous reports have shown that hiPSC-derived kidney organoids contain segmented structures with podocytes, proximal tubules, and distal tubules in nephron-like arrangements. They show a native genomic context and cell-type heterogeneity within the kidney [18,19,20]. In addition, kidney organoids from genetically modified or patient-derived hiPSCs can successfully recapitulate the phenotype of various genetic kidney diseases [18,19,21,22,23,24].

Based on the above background, the aim of this study was to establish a *FAN1*-deficient kidney disease model using an hiPSC-derived kidney organoid system. For this, we created *FAN1*-mutant hiPSCs using KIN patient-derived peripheral blood mononuclear cells and performed *FAN1* gene editing using CRISPR/Cas9 in wild-type hiPSCs. We then differentiated them into kidney organoids using a well-established protocol [21,22,23,24,25]. After that, we investigated whether the disease phenotype of KIN was recapitulated in *FAN1*-mutant kidney organoids in comparison with wild-type kidney organoids.

## 2. Materials and Methods

### 2.1. FAN1 Gene Sequencing

Genomic DNA was extracted from peripheral blood leukocytes using a QIAmp DNA Mini Kit (Qiagen, Hamburg, Germany) according to the manufacturer’s protocol. PCR was carried out using designed primers that covered all exons and flanking intronic sequences of the *FAN1* gene. PCR amplicons were bi-directionally sequenced using a Big Dye terminator v3.1 cycle sequencing kit (Applied Biosystems, Foster City, CA, USA) on an ABI PRISM 3100 Genetic Analyzer (Applied Biosystems, Foster City, CA, USA). Chromatograms were analyzed using Sequencher software version 5.0 (Gene Codes, Ann Arbor, MI, USA).

### 2.2. Gene Editing Using CRISPR/Cas9

To generate *FAN1* gene mutant hiPSCs in healthy wild-type control hiPSCs (WTC-11) using the CRISPR/Cas9 system, single guide RNA (sgRNA) was designed using the Macrogen CRISPR/Cas9 technology service (Gasan-dong, Geumcheon-gu, Seoul, Korea). sgRNA and Cas9 protein were added via electroporation using NEPAGENE (NEPA21, NepaGene Co., Ltd., Chiba, Japan) as the ribonucleoprotein (RPN) complex. Briefly, the RNP complex was formed by mixing 30 μg of Cas9 protein with 25 μg of sgRNA and incubating the mixture at room temperature for 10 min. The RNP complex was then electroporated to 1 × 10^6^ hiPSCs with 100 μL of Opti-MEM Medium (#31985062; Thermo Fisher Scientific, Grand Island, NY, USA). After transfecting for at least 72 h, the Genomic DNA Extraction Kit (#MN740230.250; Macherey-Nagel, Allentown, PA, USA) was used for genomic DNA (gDNA) extraction according to the manufacturer’s protocol. To determine gene editing efficiency and the exact sequence edited, we utilized an IN/DEL analysis service for CRISPR validation (#ATC-0120; Bioneer Corp., Daedeok-gu, Daejeon, Korea).

### 2.3. hiPSC Cell Culture

Human iPSCs were cultured under feeder cell-free conditions in mTeSR1 (#85850; STEMCELL Technologies Inc., Vancouver, BC, Canada) on 1.25% Matrigel^®^ hESC Qualified Matrix-coated (#354277; BD Bioscience, Bedford, MA, USA) 6-well tissue culture plates. Cells were split before reaching 100% confluence using TrypLE Express (#12604-013; Gibco, Grand Island, NY, USA) and replated in mTeSR1 medium supplemented with 10 μM ROCK inhibitor Y-27632 (#1293823; Biogems, Westlake Village, CA, USA). All cell lines were cultured at 37 °C with 5% CO_2_ in a humidified atmosphere.

### 2.4. Tri-Lineage Differentiation

Trilineage differentiation was performed as described previously [12,24] using a StemMACS™ Trilineage Differentiation Kit (#130-115-660; Miltenyi Biotec, Gaithersburg, MD, USA). Differentiated germ layers were confirmed by staining with antibodies: anti-PAX6 antibody for the ectoderm, anti-SM22A antibody for the mesoderm, and anti-FOXA2 antibody for the endoderm.

### 2.5. Kidney Organoid Differentiation from hiPSCs

hiPSCs were differentiated into kidney organoids using the previously published protocol [21,22,23,24,25]. 

Kidney organoids were cultured in this medium until collection on day 21. They were treated with 20 nM Mitomycin C (MMC, #M0503, Sigma-Aldrich, St. Louis, MO, USA) on day 19 of differentiation for either 24 h or 48 h.

### 2.6. Flow Cytometry

Dissociated hiPSCs or kidney organoids using TrypLE Express were washed twice with FACS buffer (phosphate-buffered saline (PBS) containing 1% bovine serum albumin and 10 mM sodium azide), then permeabilized for 30 min using flow cytometry fixation and permeabilization solution (#554714; BD Biosciences, Torreyana Road, San Diego, CA, USA).

Cells were stained with stage-specific embryonic antigens SSEA-4 (#813-70; 1:100, Santa Cruz Biotechnology, Dallas, TX, USA) or TRA-1–81 (#sc-21706; 1:100; Santa Cruz Biotechnology, Dallas, TX, USA) surface antibodies for 30 min. Intracellular staining for NANOG (#sc-293121;1:100, Santa Cruz Biotechnology, Dallas, TX, USA) was performed by means of sequential incubation with fixation and permeabilization solutions (#554722; BD Bioscience).

The cells were then incubated with a FITC-conjugated secondary antibody (#sc-2010; 1:100; Santa Cruz Biotechnology, Dallas, TX, USA) and subsequently analyzed using IgG1 isotypes, as a control for FITC (#25-4714-42; 1:100; eBioscience, Science Center Drive San Diego, CA, USA). Kidney organoids were then stained with fluorescein-conjugated Lots Tetragonolobus Lectin (LTL) antibody (#FL1321, Vector Laboratories, Burlingame, CA, USA), PE-conjugated podocalyxin (PODXL) monoclonal antibody (#12-8873-42; Life Technologies, Carlsbad, CA, USA), APC-conjugated E-Cadherin (ECAD) antibody (#FAB18381A; R&D Systems, Minneapolis, MN, USA), and Fixable Viability Dye eFluor506 (Healthy live cell marker, #65-0866; Life Technologies). Cells were then examined using a FACS Canto II (BD Biosciences), and data were analyzed with FlowJo software version 10.8.1. (Becton, Dickinson & Company, Ashland, OR, USA).

### 2.7. RT-PCR

Total RNA was extracted from cells using RNA-Bee (#CD-105B; Tel-Test, Friendswood, TX, USA) following the manufacturer’s instructions. About 1 μg of total RNA was then employed for cDNA synthesis with a cDNA synthesis kit (#DYRT1120; Dyne Bio Inc, Seongnam-si, South Korea). PCR was carried out with AccuPower PCR premix (#K2016, Bioneer Corp) using the primer set for human *FAN1* (Forward, 5′-CTC AGG GTT GTC TCC TCG TT-3′, Reverse, 5′ TTT GTC TTT GGT GGT GGT GAC-3′). This yielded a product of 589 base pairs (bps). The optimized reaction conditions for PCR amplification were as follows: initial denaturation at 94 °C for 5 min; 35 cycles of denaturation at 94 °C for 30 s, annealing at 60 °C for 30 s, and extension at 72 °C for 30 s; and a final elongation step at 72 °C for 10 min. PCR products were run on 1.2% agarose in Tris-Borate-EDTA buffer at 100 V for 30 min. The expression levels of *FAN1* were compared to those of glyceraldehyde 3-phosphate dehydrogenase (GAPDH, a housekeeping gene with functions in glycolysis). The following forward and reverse primers for GAPDH were used: 5′-CTC AGG GTT GTC TCC TCG TT-3′ and 5′-ATG GTC TCA ACA CCT GCT TC-3′. These yielded a product of 93 bps.

### 2.8. Immunofluorescence

hiPSCs or kidney organoids were washed once with PBS, fixed with 4% paraformaldehyde for 10 min at 4 °C, and then blocked with 5% normal donkey serum in PBS-T (0.3% Triton X-100 in PBS) for 1 h at room temperature (RT). hiPSCs or kidney organoids were incubated with the following primary antibodies at 4 °C overnight: anti-NANOG (#sc-293121; 1:100, Santa Cruz Biotechnology, Dallas, TX, USA), anti-SSEA-4 (#MAB4304; 1:100, Millipore Sigma, Burlington, MA, USA), anti–TRA-1-81 (#MAB4381; 1:100, Millipore Sigma), anti-PAX6 (#sc-81649; 1:20, Santa Cruz Biotechnology, Dallas, TX, USA), anti-SM22A (#sc-53932; 1:40, Santa Cruz Biotechnology, Dallas, TX, USA), anti-FOXA2 (#sc-374376; 1:50, Santa Cruz Biotechnology, Dallas, TX, USA), biotinylated Lotus Lectin antibody (LTL; #B-1323;1:100, Vector Laboratories), anti-E-cadherin (ECAD; #ab11512; 1:50, Abcam, Cambridge, UK, #610181; BD Biosciences), anti-podocalyxin (PODXL; #BAF1658; 1:100, R&D Systems), and anti-Ki67 (#ab15580; 1:50, Abcam). Cells were then stained with secondary antibodies: Alexa Fluor 488-donkey anti-mouse IgG (#A32766; 1:250, Invitrogen, Camarillo, CA, USA), Fluorescein (DTAF) Streptavidin (016-010-084; Jackson Immuno Research, West Grove, PA, USA), Alexa Fluor 647-donkey anti-goat IgG (#A32849; 1:250; Invitrogen, Camarillo, CA, USA), Alexa Fluor 647-donkey anti-mouse IgG (#A-31571; Invitrogen, Camarillo, CA, USA), Streptavidin conjugate Cyanine5 (Cy5) (#SA1011; Invitrogen, Camarillo, CA, USA), Cyanine3(Cy3)-streptavidin (#016-160-084; 1:1000, Jackson Immuno Research, West Grove, PA, USA), Alexa Fluor 488-donkey anti-rat IgG (#A48269; 1:250, Invitrogen, Camarillo, CA, USA), and Cy3-conjugated donkey anti-rabbit (#711-165-152; 1:1000, Jackson Immuno Research). Nucleic acid staining was performed via incubation with 40,6-diamidino-2-phenylindole (DAPI; #10236276001; 1:5000, Roche, Basel, Switzerland) for 30 min at RT. Images were obtained using a Zeiss LSM700 confocal microscope (Carl Zeiss MicroImaging GmbH, Jena, Germany).

### 2.9. Immunoblot Analysis

hiPSCs or kidney organoids were lysed in protein extraction solution (10 mM Tris (pH 7.5) containing 1% sodium dodecyl sulfate (SDS) and 1 mM NaVO4). Equal amounts of protein were subjected to immunoblotting analysis with primary antibodies (anti-FAN1 (#ab95171; 1:500, Abcam), anti-H2A.X (#2595; 1:500, Cell signaling, Danvers, MA, USA), and β-actin (#3700; 1:2000, Cell signaling)). Signals were detected using the enhanced chemiluminescence system (#WSE-7110; ATTO Corp., Tokyo, Japan). Quantification of relative densities was performed by setting the control group at 100%. Densities were normalized to those of β-actin bands (Quantity One, v.4.4.0; Bio-Rad Laboratories, Hercules, CA, USA).

### 2.10. Statistical Analyses

All data are presented as the mean ± standard error (SE) of at least three independent experiments. One-way ANOVA works by comparing the differences among group means with the pooled standard deviations of the groups using Prism software (version 7.03 for Windows; GraphPad, La Jolla, CA, USA). Statistical significance was set at *p* < 0.05.

## 3. Results

### 3.1. Summary of Clinical Features of a Patient with Karyomegalic Interstitial Nephritis

**Figure 1:**
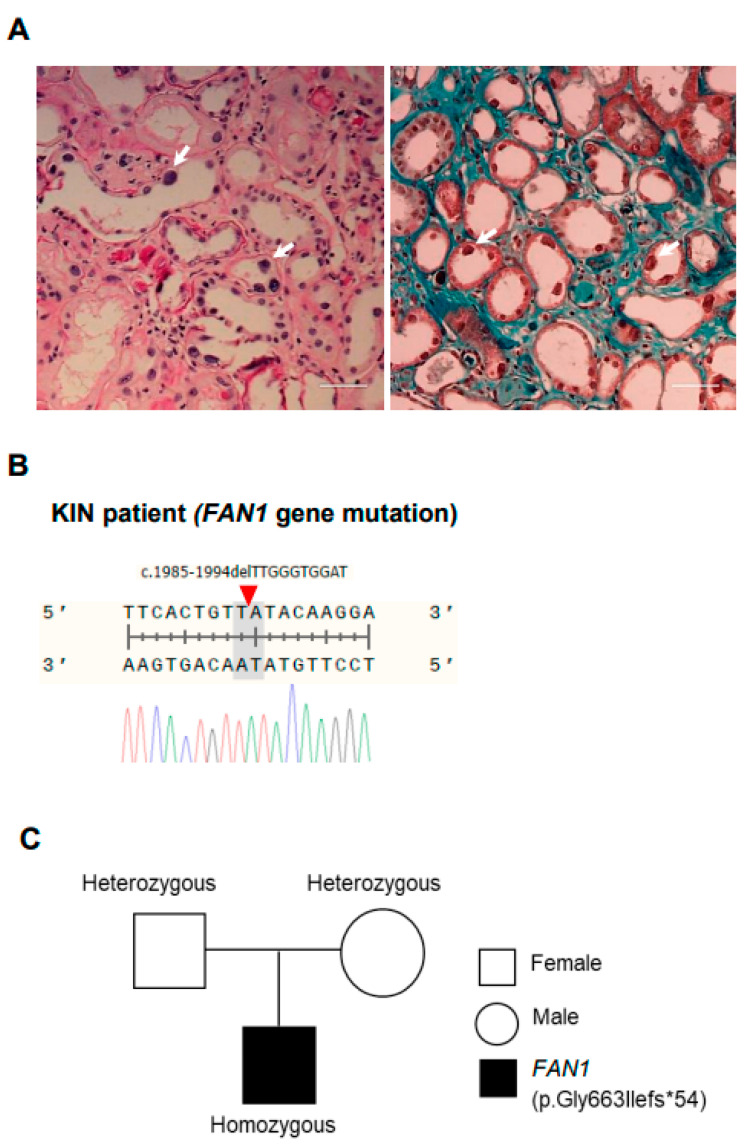
***FAN1* gene mutation in a patient with karyomegalic interstitial nephritis (KIN).** (**A**) Hematoxylin and eosin (H&E) staining of kidney tissues of a patient with karyomegalic interstitial nephritis. Scale bar = 100 μm. White arrows in A point to karyomegaly. (**B**) Patient PBMC with KIN showing *FAN1* gene mutation on deletion c.1985-1944delTTGGGTGGAT on 15q13.3. (**C**) Pedigree of a family showing individuals affected by karyomegalic interstitial nephritis resulting in the appearance of p.Gly663llefs*54.

A 42-year-old woman visited our nephrology clinic because of impaired renal function. She had no familial history of kidney disease. Her serum creatinine level was 2.06 mg/dL with an estimated glomerular filtration rate of 26 mL/min/1.73 m^2^ as calculated using the CKD-EPI (chronic kidney disease-Epidemiology Collaboration) formula. Abdominal ultrasonography revealed a relatively small right kidney, measured at 7.4 × 3.6 cm against 9.2 × 4.3 cm for the left one. There was no hepatomegaly or splenomegaly. We performed a kidney biopsy to investigate the unexplained renal function impairment and proteinuria. The biopsy tissue specimen revealed that 50% of the glomeruli (10 out of 20) were globally sclerotic. The remainder appeared normal without deposits in a immunofluorescence study. Patchy but severe interstitial fibrosis and tubular atrophy were detected (Figure 1A). Notably, numerous tubular cells in both the cortex and medulla showed nuclear enlargement with irregular outlines, hyperchromatic aspect, and prominent nucleoli, but without intra-nuclear inclusions (arrows in Figure 1A). These findings strongly suggested karyomegalic interstitial nephritis (KIN), a hereditary cause of chronic interstitial nephritis. With suspension of KIN, we performed Sanger sequencing of the *FAN1* gene. The results revealed a homozygous frameshifting deletion mutation (c.1985_1994del10 encoding p.Gly663Ilefs*54) in *FAN1* (Figure 1B). This mutation has been reported in dbSNP as rs751551936. We also conducted Sanger sequencing for the proband’s parents for segregation analysis and detected one heterozygous mutation in each parent (Figure 1C). With a confirmed diagnosis of KIN due to *FAN1* gene mutation, we generated hiPSCs using PBMCs isolated from this patient as previously reported (CMCi001-A (KIN patient-hiPSCs)) [26].

### 3.2. Generation of FAN1 Gene Mutation hiPSC Using CRISPR/Cas9 System

**Figure 2:**
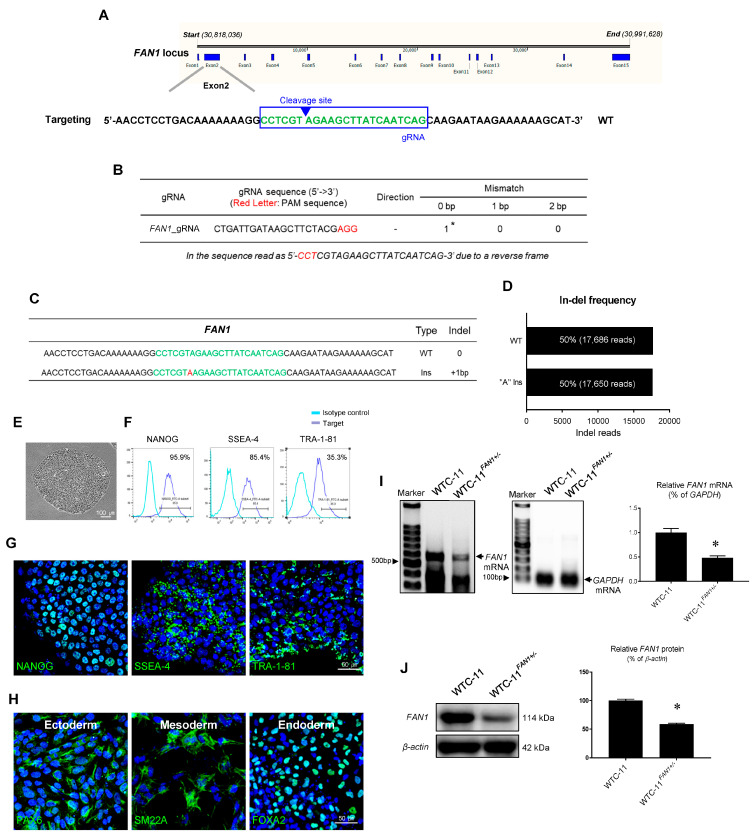
**Establishment of CRISPR/Cas9 ribonucleoproteins (RNP)-mediated *FAN1* gene editing in WTC-11 hiPSCs.** (**A**) Target site for guide RNA (gRNA) targeting *FAN1*. Target sites are indicated by a green color in exon 2 of the *FAN1* gene. The downward-pointing arrowhead indicates the position of the canonical cut site and predicted specificity based on the number and distribution of homoeologous SNPs at the corresponding target site/PAM. (**B**) PAM sequences (5′-NGG-3′) in target site are indicated by red letters. The table shows no mismatched number with gRNA. The asterisk indicates on-target gRNA. (**C**) Indel sequences after transfecting WTC-11 hiPSCs with gRNA using the CRISPR/Cas9 RNP method. Indel sequences are indicated by red letters (a green color indicates a target site). (**D**) Read number of In-del frequency. Indel of 1bp (A ins) in the target sequence read of about 50%. (**E**) Morphology of *FAN1*-gene-edited WTC-11 iPSC (WTC-11*^FAN1+/−^*). (**F**,**G**) Flow cytometry analysis and immunofluorescence image of cells expressing NANOG, SSEA-4, and TRA-1-81 in WTC-11*^FAN1+/−^* hiPSCs. Scale bar = 50 μm. (**H**) Immunofluorescence staining of three germ layer markers. Ectoderm, mesoderm, and entoderm differentiation were detected using PAX4, SM22a, and FOX2A, respectively. Scale bar = 50 μm. (**I**) Expression analysis via RT-PCR of *FAN1* and *GAPDH* in WTC-11 and WTC*^FAN1+/−^* hiPSCs. All expression levels were normalized against *GAPDH* expression level. (**J**) Expression analysis via immunoblot of FAN1 and *β-actin* in WTC-11 and WTC*^FAN1+/−^* hiPSCs. All expression levels were normalized against the *β-actin* expression level. Data are presented as mean ± standard error. *, *p* < 0.05 vs. WTC-11 iPSC.

Given that our current case of a KIN patient highlights the critical role of *FAN1* in CKD induced by a 15q13.3 mutation, we investigated the consequences of *FAN1* gene mutation in hiPSCs. Analysis of the *FAN1* locus (ENSG00000198690, NC_000015.10 [30,818,036…30,991,628]) revealed that exon 2 is the common coding exon shared by known transcript variants. We first designed a CRISPR guide RNA (gRNA) targeting exon 2 of the *FAN1* locus (cut site: chr15[+30,818,889: −30,818,890], Figure 2A) with the lowest combined off-targeted score. The gRNA was incubated with recombinant Cas9 protein to generate CRISPR/Cas9-gRNA RNP complexes with the WTC-11 hiPSC line (WTC-11). The result of target region sequencing after transfecting WTC-11-hiPSCs with the RNP complex showed that the *FAN1* gene was edited with an in-frame mutation +1bp (Adenine) between cleavage site showing a 50% of in-del frequency heterozygous for this mutation (Figure 2C,D). The in-frame mutation was confirmed by analyzing protein sequences translated from the DNA sequences, and deletion of 49 amino acids at the beginning of the *FAN1* mRNA was found.

### 3.3. Characterization of FAN1 Gene Edited WTC-11 hiPSC

The generated *FAN1*-edited hiPSCs (WTC-11*^FAN1+/^^−^*) displayed typical pluripotent stem cell-like morphology and expressed pluripotent markers such as NANOG, SSEA-4, and TRA-1-81 according to flow cytometry and immunofluorescence (Figure 2E–G). Tri-lineage differentiation assays demonstrated that WTC-11*^FAN1+/^^−^* hiPSCs were successfully differentiated into ectoderm, mesoderm, and endoderm, as confirmed by immunostaining with PAX6, SM22A, and FOXA2, respectively (Figure 2H).

Next, we compared mRNA and protein expression levels of *FAN1* in the generated WTC-1*^FAN1+/^^−^* and WTC-11 hiPSC. Using RT-PCR analysis, we found that the mRNA level of *FAN1* was significantly attenuated in WTC-1*^FAN1+/^^−^* hiPSCs compared to WTC-11-hiPSCs (0.48 ± 0.045 vs. 1 ± 0.084, *p* < 0.05 vs. WTC-11-hiPSC group) (Figure 2I). Consistently, the relative expression level of FAN1 was also reduced in the WTC-1*^FAN1+/^^−^* hiPSCs group compared to the WTC-11 group (58.8 ± 1.5 vs. 100 ± 2.4, *p* < 0.05 vs. WTC-11 hiPSC group) (Figure 2J).

### 3.4. Differentiation of FAN1-Mutant hiPSCs into Kidney Organoid

**Figure 3:**
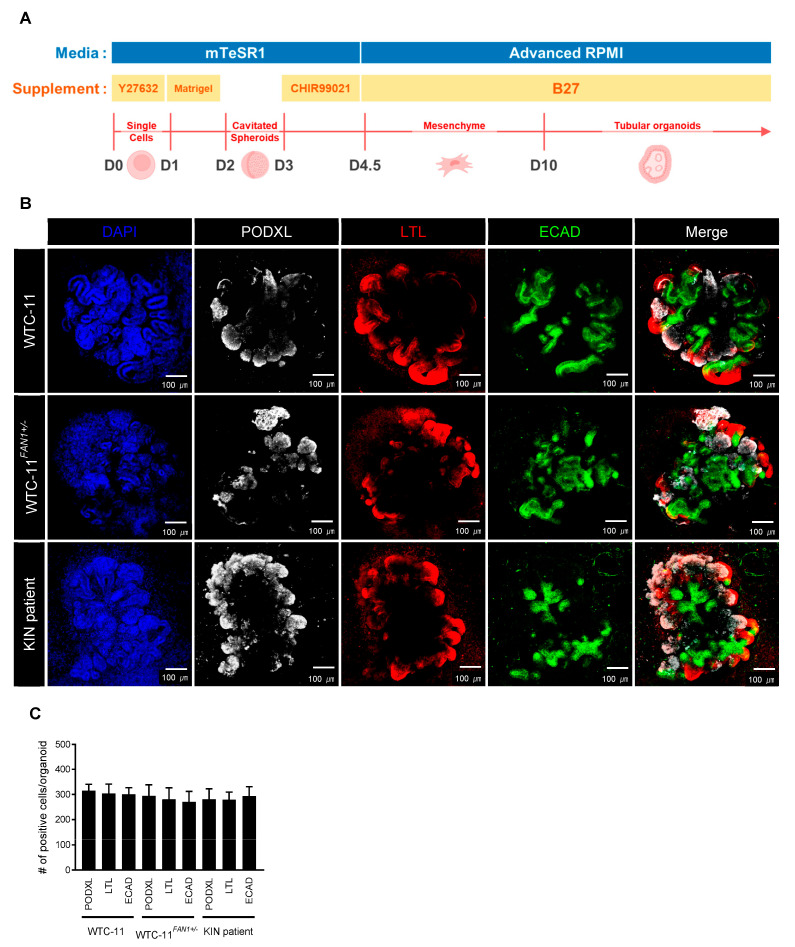
**Differentiation of kidney organoids from WTC-11, WTC-11*^FAN1+/−^*, and KIN patient hiPSCs.** (**A**) Schematic timeline of the hiPSC differentiation protocol. (**B**) Representative immunofluorescence images of podocalyxin (PODXL), lotus tetragonolobus lectin (LTL), and e-cadherin (ECAD) kidney organoids from WTC-11, WTC-11*^FAN1+/−^*, and KIN patient, respectively. (**C**) Quantification of PODXL, LTL or ECAD-positive cells per organoid in each group. Scale bar = 50 or 100 μm.

We differentiated both *FAN1*-mutant hiPSCs (KIN patient hiPSCs and WTC-1*^FAN1+/^^−^*) and WTC-11 hiPSC lines into kidney organoids using a previously established adherent culture protocol (Figure 3A) [21,22,23,24]. At 21 days after plating, typical segmented tubular structures were detected. Immunofluorescence staining and examination under confocal microscopy revealed that all three hiPSC lines were successfully differentiated into kidney organoids without structural deformities. They all expressed markers for various components of nephron structure, including podocalyxin (PODXL) in glomerular epithelial cells, lotus tetragonolobus lectin (LTL) in the proximal tubules, and e-cadherin (ECAD) in the distal tubule, in appropriately patterned and contiguous segments (Figure 3B). In addition, we counted the numbers of cells expressing each marker, and found that there was no significant difference among the groups, as shown in Figure 3C.

### 3.5. Effect of Mitomycin C treatment in Kidney Organoids from WTC-11, WTC-11^FAN1+/−^, and KIN Patient hiPSCs

**Figure 4:**
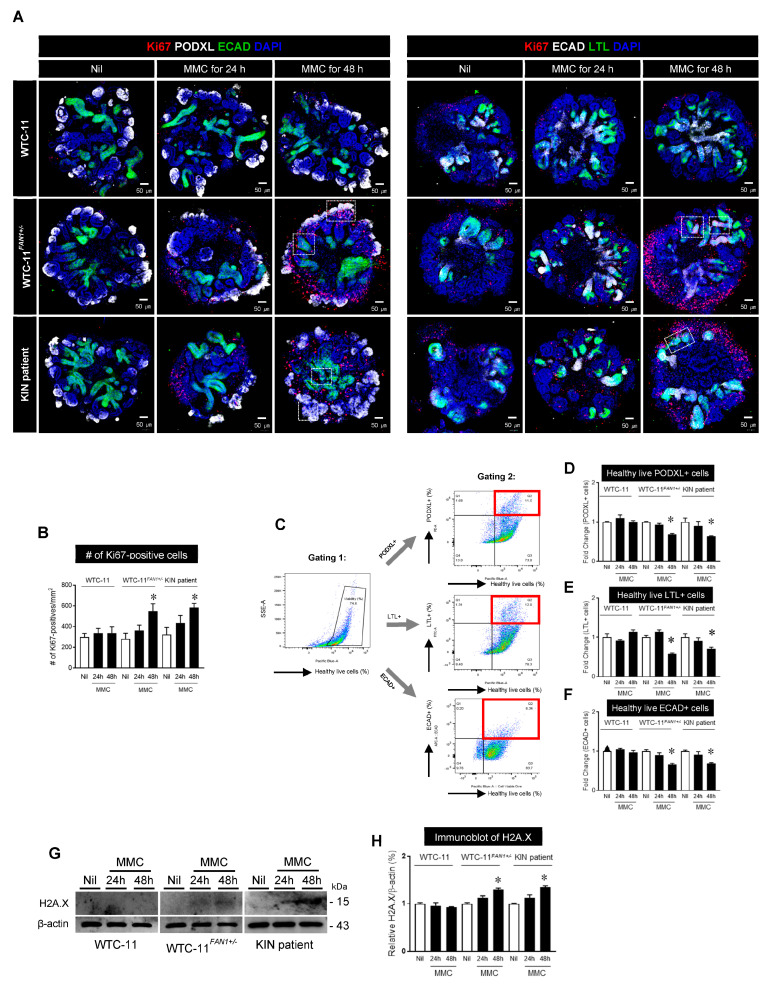
**Effect of mitomycin C (MMC) treatment in kidney organoids from WTC-11, WTC-11*^FAN1+/−^*, and KIN patient hiPSCs.** (**A**) Each kidney organoid was treated with 20 nM MMC. After 24 h or 48 h of incubation, each kidney organoid was stained with Ki67 antibody. Images with DAPI show nuclei positive for the Ki67 antibody. Scale bar = 50 or 100 μm. (**B**) Quantification of Ki67-positive cells in kidney organoids from WTC-11, WTC-11*^FAN1+/−^*, and KIN patient hiPSCs. (**C**) Flow cytometry gating strategy illustrating a viable cell population being subgated to the level of podocalyxin+ (PODXL), lotus tetragonolobus lectin+ (LTL), or e-cadherin+ (ECAD) in kidney organoid cells from WTC-11, WTC-11*^FAN1+/−^*, and the KIN patient hiPSCs, respectively, after treatment with MMC for 24 h or 48 h. (**D**–**F**) Quantification of percentage of viable cells in PODXL+, LTL+, or ECAD+ in cells from each kidney organoid. (**G**,**H**) Immunoblot analysis and its quantification of H2A.X in kidney organoids from WTC-11, WTC-11*^FAN1+/−^*, and KIN patient hiPSCs after treatment with MMC for 24 h or 48 h. Data were normalized against the β-actin expression level. All data are presented as mean ± standard error. *, *p* < 0.05 vs. Nil group or 24 h MMC group.

Expression of Ki67: For KIN disease modeling using *FAN1*-mutant kidney organoids, we first determined mitomycin C (MMC) sensitivity in the kidney organoid derived from WTC-11, WTC-11*^FAN1+/^^−^*, and KIN hiPSCs by analyzing Ki67 expression (Figure 4A,B). The expression of Ki67 in the WTC-11 kidney organoids did not change after MMC treatment for 24 h or 48 h. On the other hand, the number of Ki67-positive cells significantly increased in the 48 h MMC treatment group for both *FAN1*-mutant kidney organoids (WTC-11*^FAN1+/^^−^* and KIN patient) compared with each of the corresponding Nil groups (337 ± 62 vs. 300 ± 34 in the WTC-11 group, *p* < 0.05 vs. Nil; 549 ± 71 vs. 281 ± 54 in the WTC-11*^FAN1+/^^−^* group, *p* < 0.05 vs. Nil; 585 ± 40 vs. 323 ± 70 in the KIN patient group, *p* < 0.05 vs. Nil).

Cell viability using flow cytometric analysis: Next, we investigated whether increased MMC sensitivity of the kidney organoids derived from *FAN1*-mutant hiPSCs could affect cell viability using flow cytometry analysis. After treating each kidney organoid with MMC and dissociating them into single cells, these cells were stained with antibodies against PODXL, LTL, and ECAD, and a cell viability dye. Initially, we first gated the positive area for viable cells. We then re-gated it using specific nephron markers such as PODXL, LTL, or ECAD as shown in Figure 4C. The percentage of viable cells among PODXL, LTL, or ECAD-positive cells was significantly reduced after MMC treatment for 48 h in the group of WTC-11*^FAN1+/^^−^* and KIN patient hiPSCs compared to that in the WTC-11 group (viable PODXL+ cells: 0.69 ± 0.02% in WTC-11*^FAN1+/^^−^*, 0.64 ± 0.01% in the KIN patient, and 1 ± 0.03% in WTC-11; viable LTL+ cells: 0.58 ± 0.02% in WTC-11*^FAN1+/^^−^*, 0.71 ± 0.04% in the KIN patient, and 1.14 ± 0.05% in WTC-11; viable ECAD+ cells: 0.66 ± 0.03% in WTC-11*^FAN1+/^^−^*, 0.69 ± 0.02% in the KIN patient, and 0.97 ± 0.05% in WTC-11) (Figure 4D–F).

Expression of H2A.X: We also evaluated the expression of H2A histone family member X (H2A.X) to compare DNA double-strand breaks and DNA damage in each kidney organoid group after MMC treatment. Immunoblotting and subsequent quantification of H2A.X revealed that the abundance of the H2A.X protein in WTC-11 did not differ among the Nil, 24 h, and 48 h MMC treatment groups. However, a 48 h MMC treatment significantly elevated the level of H2A.X protein both the WTC-11*^FAN1+/^^−^* and KIN patient groups compared to the corresponding Nil or 24 h MMC groups (1.30 ± 0.03 in the WTC-11*^FAN1+/^^−^* and 1.35 ± 0.03 in the KIN patient group vs. 0.93 ± 0.01 in WTC-11, *p* < 0.05 vs. WTC-11). 

## 4. Discussion

In this study, we generated hiPSCs derived from a KIN patient as well as *FAN1*-edited hiPSCs. These cells were then differentiated into kidney organoids. Compared to wild-type kidney organoids, both types of *FAN1*-mutant kidney organoids displayed decreased levels of healthy viable cells and increased levels of DNA damage markers under when treated with MMC. Our study suggests that the hiPSC-derived kidney organoid system can effectively recapitulate the phenotype of *FAN1*-deficient kidney disease. It could be used as a valuable platform for future research investigating the relationship between DNA damage and the progression of CKD in humans.

Many previous studies have suggested that CKD can rise from incomplete resolution of DNA damage in the kidney following exposure to geno-toxins or environmental toxins [27]. KIN has been linked to mutations in the *FAN1* gene [1], which plays a role in the DNA damage response pathway, particularly in the kidney. This sheds new light on the potential link between defective DNA repair and CKD progression. In this regard, using KIN as a model for *FAN1*-deficient kidney disease can be a valuable platform for investigating the mechanisms by which CKD develops due to defective DNA repair. It also could be used to develop therapies that could block the progression of CKD by targeting these mechanisms. Previous studies have successfully modeled *FAN1*-deficient kidney disease using zebra fish and mice, demonstrating increased DNA damage response (DDR), apoptosis, and karyomegaly [1,2,3,8]. However, no studies have yet reported on the modeling of *FAN1*-deficient kidney disease using an hiPSC-derived kidney organoid system.

In this regard, our first aim was to investigate whether KIN patient-derived or *FAN1*-gene-edited hiPSC-derived kidney organoids could exhibit the phenotype of KIN. To investigate the KIN phenotype in kidney organoids, we assessed the percentage of viable cells using dye labeling and the expression of DNA damage markers under treatment conditions with MMC. It is well known that KIN and polyploidization are associated with exposure to high levels of environmental toxins or chemotherapeutics that damage renal cells [28,29]. Therefore, progressive kidney failure in *FAN1*-deficient individuals with KIN can be induced by defects in genome maintenance, causing cellular damage, which does not typically occur in individuals with a functional *FAN1* gene. In this regard, we treated kidney organoids derived from wild-type hiPSCs and *FAN1*-mutant hiPSCs with mitomycin C and compared cell viability and expression levels of DNA damage markers. Previous studies have shown that cell death due to *FAN1* deficiency is caused by apoptosis through the activation of DDR signaling [2]. Thus, we investigated the number of Ki-67 positive cells and expression levels of H2A histone family member X (H2A.X), a sensitive marker of DNA double-strand breaks that has been used to detect DNA damage in human tissues [30,31]. As a result, we found that the number of Ki-67-positive cells and the expression of H2A.X were increased in both *FAN1*-mutant kidney organoids accompanied by a low percentage of healthy live cells, but no such changes were observed in WTC-11-kidney organoids. Taken together, our results suggest that the kidney organoid system could recapitulate the disease phenotype in terms of a vulnerable state in response to DNA damage.

Experiments concerning KIN using human cell lines have mostly been performed using kidney proximal tubular epithelial cells [1,2,8]. The effect of *FAN1* deficiency on other cell types has not yet been fully investigated. Therefore, the advantage of research using kidney organoids is that effects of *FAN1* deficiency can be confirmed not only in proximal tubule cells, but also in various cells constituting the nephron. [22,24] As a result, we found co-localization of Ki-67 not only in proximal tubular epithelial cells, but also in podocyte and distal tubular cells, as shown in Figure 4A. In addition, healthy live cells were decreased in all nephron segmental cells in flowcytometry (Figure 4C,D). Considering the process by which genetic or environmental toxins enter the body and are filtered out through the kidney, renal proximal tubular epithelial cells are likely to be the most affected by these toxins. That is why human and mouse kidney specimens display karyomegalic mainly in the renal proximal tubular epithelial cells. However, this does not imply that other cells constituting the kidney are unaffected by *FAN1* deficiency. This study using a kidney organoid system suggests that when similarly exposed to genetic or environmental toxins, other cell types in the kidney also display decreased cell viability through DDR activation.

In this study, we used both KIN patient-derived hiPSCs and *FAN1* gene-edited hiPSCs with the CRISPR/Cas9 system. Both systems have a compensative role for modeling genetic kidney disease [32]. In most genetic disorders, disease phenotype can be different according to the mutation type in affected individuals [33,34,35]. Indeed, in our previous study, we found that disease severity varied significantly among kidney organoids based on the mutation type in patients from which they originated [22]. To reliably inactivate target gene and ensure robust disease modeling, applying target gene-edited hiPSC can be preferable. However, the application of the CRISPR/Cas9 technique carries a risk of inducing off-target effects on non-target genes [36,37]. In this regard, the use of both patient-derived and gene-edited hiPSCs is recommended for reliable disease modeling with the kidney organoid system.

This study has some limitations. The kidney organoids developed via the protocol used in this study represent an immature form of the kidney, most closely resembling the third trimester human fetal kidney [21,25]. This suggests that the modeling of adult-onset kidney diseases can be limited with this organoid differentiation protocol. In KIN, DNA damage and insufficient DNA repair processes occur over a long time period, accumulate, and ultimately manifest as karyomegaly. Therefore, reproducing karyomegaly in this immature in vitro kidney model could be very difficult. This raises the question of whether kidney organoids might not be appropriate for modeling KIN. Nevertheless, we demonstrated increased sensitivity to DNA damage in each cell type constituting kidneys in *FAN1*-mutant kidney organoids, even though we failed to induce karyomegaly. We consider this study as a proof-of-concept for modeling KIN using the kidney organoid system. In the future, improvements in the cellular maturation levels of the kidney organoids could be achieved using more advanced protocols, such as refining culture conditions or introducing a microfluidic system. This could expand the repertoire of kidney diseases amenable to disease modeling using the kidney organoid system.

## 5. Conclusions

In conclusion, we found that phenotypes associated with KIN, such as increased sensitivity and cell death to DNA damage, were successfully recapitulated by a kidney organoid system generated using both KIN patient-derived and *FAN1*-edited hiPSCs through the CRISPR/Cas9 technique. Although karyomegaly in *FAN1*-deficient kidney cells is thought to arise from DNA polyploidization due to mitotic errors, and result in cell damage, the effects of *FAN1* deficiency on the intracellular molecular pathway have not been fully elucidated yet. We expect that this *FAN1*-mutant kidney organoid model will serve as a valuable platform for future research aiming to unveil the molecular mechanisms associated with the development of CKD caused by DNA damage and incomplete repair.

## Data Availability

The datasets generated and/or analyzed during the current study are available from the corresponding author upon reasonable request.
